# Development and validation of cancer-associated fibroblasts-related gene landscape in prognosis and immune microenvironment of bladder cancer

**DOI:** 10.3389/fonc.2023.1174252

**Published:** 2023-06-15

**Authors:** Meng Zhang, Junlong Zhu, Pan Zhang, Lingxun Li, Min Min, Tinghao Li, Weiyang He

**Affiliations:** ^1^ Department of Urology, The First Affiliated Hospital of Chongqing Medical University, Chongqing, China; ^2^ Department of Urology, The Third Hospital of Mianyang, Sichuan Mental Health Center, Mianyang, China

**Keywords:** cancer-associated fibroblasts (CAF), prognosis, immune microenvironment, bladder cancer, tumor microenvironment

## Abstract

**Backgrounds:**

Bladder cancer (BLCA) is one of the most prevalent cancers of the genitourinary system, the clinical outcomes of patients with BLCA are bad, and the morbidity rate is high. One of the key components of the tumor microenvironment (TME) is cancer-associated fibroblasts (CAFs) which are critically involved in BLCA tumorigenesis. Previous studies have shown the involvement of CAFs in tumor growth, cancer progression, immune evasion, angiogenesis, and chemoresistance in several cancers such as breast, colon, pancreatic, ovarian, and prostate cancers. However, only a few studies have shown the role of CAFs in the occurrence and development of BLCA.

**Methods:**

We have retrieved and merged the data on RNA-sequencing of patients with BLCA from databases including “the Cancer Genome Atlas” and “Gene Expression Omnibus.” Next, we compared the differences in CAFs-related genes (CRGs) expression between normal and BLCA tissues. Based on CRGs expression, we randomly divided patients into two groups. Next, we determined the correlation between CAFs subtypes and differentially expressed CRGs (DECRGs) between the two subtypes. Furthermore, the “Gene Ontology” and “Kyoto Encyclopedia of Genes and Genomes pathway” enrichment analyses were conducted to determine the functional characteristics between the DECRGs and clinicopathology.

**Results:**

We identified five genes (*POF1B, ARMCX1, ALDOC, C19orf33*, and *KRT13*) using multivariate COX regression and “Least Absolute Shrinkage and Selection Operator (LASSO) COX regression analysis” for developing a prognostic model and calculating the CRGs-risk score. The TME, mutation, CSC index, and drug sensitivity were also analyzed.

**Conclusion:**

We constructed a novel five- CRGs prognostic model, which sheds light on the roles of CAFs in BLCA.

## 1 Introduction

Bladder Cancer (BLCA) is the most prevalent cancer of the genitourinary system (1). In 2020, approximately 81,400 new incidences and 17,980 new BLCA-related mortalities were reported in the USA (2). BLCA can be further divided into non-muscle-invasive (NMIBC) and muscle-invasive bladder cancer (MIBC) (3). Various therapeutic strategies like surgery, intravesical chemo, radiotherapy, and immunotherapies have helped significantly improve the prognosis of patients with BLCA. However, the survival outcomes of patients with BLCA are unsatisfactory due to high genomic instability and heterogeneity in BLCA (4). Unfortunately, the 5-year overall survival (OS) rate of patients with high-grade MIBC and metastatic BLCA is only 15%. This poses a huge burden on public health and the economy globally (5). Hence, it is essential to identify the underlying mechanism for preventing BLCA and treating patients with this disease. Therefore, identifying and establishing a prognostic gene signature model for predicting the prognosis of patients with BLCA is crucial.

Along with cancer cells, the tumor microenvironment (TME) regulates and influence tumorigenesis and cancer progression (6). TME is a complex system, primarily comprising various cells like infiltrated stromal and immune cells, endothelial cells, and cancer cells. Extracellular matrix (ECM), several signaling molecules, and soluble biological factors comprise the non-cellular component of TME (7, 8). Therefore, TME has a significant impact on BLCA development. Bladder tumor microenvironment comprises several types of stromal and immune cells (9). Cancer-associated fibroblasts (CAFs) are the most abundant stromal cell type in TME (10). CAFs are an important component of the ECM of TME, which participates in cell invasion, angiogenesis, and ECM remodeling by secreting factors to promote cell invasion and enhance cell-cell interaction (11). Mounting evidence has revealed the close correlation between CAFs and poor prognosis of patients with ovarian, colon, and gastric cancers (12–14) and their underlying mechanisms. However, TME, specifically CAFs in bladder tumor microenvironment, have not been extensively studied compared to the TME of several other cancers. Therefore, exploring the biological characteristics and functions of CAFs would aid in BLCA therapeutics.

In this study, we constructed and validated the CAFs-related genes (CRGs) prognostic model using RNA-sequencing (RNA-seq) and clinical data of 958 patients with BLCA from three datasets. Next, we used bioinformatics algorithms and immunohistochemistry (IHC) to explore as well as validate the involvement of CAFs in the onset and progression of BLCA. Finally, we calculated a risk score based on CRGs expression to determine its prognostic and clinical significance in patients with BLCA. Furthermore, we explored the correlation between CRGs-risk scores, TME, and drug sensitivity. These results may provide new insights for determining the patient’s prognosis, survival, and the underlying mechanism of CAFs in BLCA.

## 2 Materials and methods

### 2.1 Data acquisition

We retrieved the RNA-seq and clinical data, copy number variation (CNV), and mutation annotation format (MAF) file of 409 BLCA and 19 normal samples from “the Cancer Genome Atlas (TCGA; https://portal.gdc.cancer.gov/)” database. The RNA-seq and clinical data of patients with BLCA from the “Gene Expression Omnibus” (GEO; GGSE13507 and GSE32894 datasets; https://www.ncbi.nlm.nih.gov/geo/) database. Next, gene expression data of patients from the TCGA-BLCA cohort, as well as the GGSE13507 and GSE32894 datasets, were merged into an entire cohort. Subsequently, we used the “Combat” algorithm to eliminate the batch effects. Finally, we included 958 patients with BLCA for subsequent analyses.

To identify CAF gene sets, we searched for “CAFs” as a keyword in a publicly available database: “Gene Set Enrichment Analysis (http://www.gsea-msigdb.org/gsea/msigdb/genesets.jsp)” and approximately 48 CRGs were screened. Additionally, 19 CRGs were collected from previous studies (15–18). Finally, 67 CRGs were obtained.

We downloaded immunohistochemistry images of tissues of BLCA and normal bladder of human origin from the “Human Protein Atlas (http://www.proteinatlas.org)” database.

### 2.2 Clustering pattern of CAFs-related genes

Based on CRGs expression, Bayesian information criterion, and consensus cluster analysis, we selected the number of clusters (k) using the “Consensus Cluster Plus” software. Next, we classified patients based on the “K-value” into two subtypes.

### 2.3 Identification and function analysis of differently expressed CAFs-related genes

We used the “linear models for microarray data (limma)” R package to identify DECRGs in tissues of normal and patients with BLCA based on the following criteria: “|log2(fold change)|>1” and “adjusted *P* < 0.05.” We identified biological functions enriched by DECRGs using the “Gene Ontology (GO)” and “Kyoto Encyclopedia of Genes and Genomes (KEGG) pathway” enrichment analysis. We used the “Gene Set Variation Analysis (GSVA)” R package to determine the activity differences of pathways or biological processes.

### 2.4 Construction and validation of the CAFs-related genes prognostic model

We determined the correlation between CRGs and patients’ prognoses from TCGA-BLCA and GEO cohorts. First, we performed the “Univariate Cox regression” analysis to identify DECRGs related to the survival of patients with BLCA using the “survival” R package. The threshold value for identifying these genes is “*P* < 0.05.” Next, we classified the patients into gene clusters A and B for further analysis. Then, we randomly categorized the patients in a 1:1 ratio into the training (n = 395) and testing (n = 395) sets. Furthermore, we employed the “least absolute shrinkage and selection operator (LASSO) regression analysis” for correcting the overfitting risk and screened DEGs. Finally, we established a CRGs prognostic model based on five CRGs. We calculated the CRGs-risk scores using the following formula: CRGs-risk scores= Σ (Expi * Coefi) Coefi is the risk coefficient, and Expi is gene expression). Finally, we classified patients with BLCA based on CRGs-risk scores into the high-risk group and the low-risk group. Next, we performed the “Kaplan-Meier (KM) survival analysis” using the “survminer” R package for comparing the OS of patients in both groups and the testing set and validated using the entire sets.

### 2.5 Evaluation of immune cell infiltration in CAFs-related genes of patients with BLCA

The “Estimation of STromal and Immune cells in MAlignant Tumor tissues using Expression data (ESTIMATE)” algorithm was employed to calculate the immune and stromal scores of patients with BLCA. The fraction scores of all tumor samples about 23 immune cell subtypes were identified by the “Cell-type Identification by Estimating Relative Subsets of RNA Transcripts (CIBERSORT)” R package (cell type identification by estimating relative subtypes of RNA transcripts). Finally, we compared the status of immune cell infiltration.

### 2.6 Evaluation of the correlation between CRGs-risk scores and TME, CSC, mutation, and drug sensitivity

The stromal and immune scores of patients with BLCA were calculated using the “ESTIMATE” algorithm. The fraction scores of 23 immune cell types in all tumor samples were calculated using the “CIBERSORT” R package, where in immune cell types were established by estimating relative levels of RNA. Next, we determined the correlation between the CRGs-risk scores and tumor mutational burden (TMB). The “maftools 2.12.0” R package was used for visualizing the results. Further, we analyzed the correlation between the cancer stem cell(CSC)-risk scores and both risk groups. The “maftools” R package was used for exploring the somatic mutations in patients from both risk groups. Finally, the “Genomics of Cancer Drug Sensitivity (GDSC; https://www.cancerrxgene.org/)” database was used to determine patients’ sensitivity to several chemotherapy drugs. Finally, we calculated the IC_50_ values using the”pRRophetic” R package.

## 3 Statistical analysis

We used the “R (version 4.2.2)” and “Perl (5.30.0.1-64bit)” software for performing all statistical analyses. All statistical tests were two-sided, and *P* < 0.05 indicated the significance level.

## 4 Results

### 4.1 Landscape of genetic and transcriptional alterations of CAF-related genes in bladder cancer

**Figure 1:**
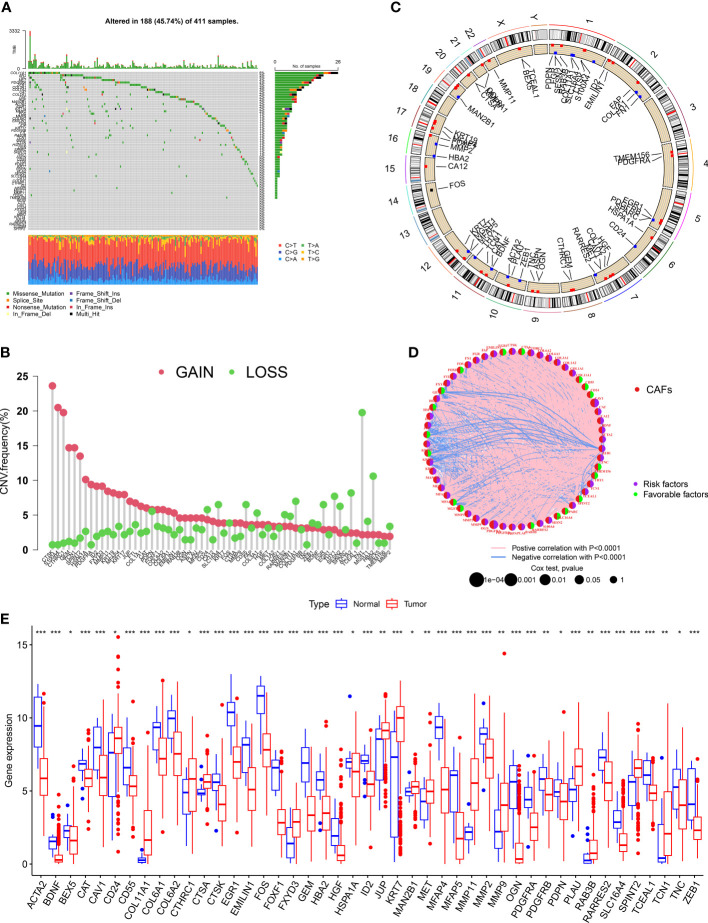
Landscape of genetic and transcriptional alterations of CAF-related genes in bladder cancer. **(A)** Somatic mutation in CRGs in patients from the TCGA- BLCA cohort. **(B)** The frequency of CNV in CRGs in patients with BLCA. **(C)**The chromosomal location of CNV in CRGs in TCGA-BLCA cohort. **(D)** The network diagram of CRGs in patients with BLCA. **(E)** The difference in the expression of 45 CRGs in tissues of normal and patients with BLCA. *P < 0.05, **P < 0.01, ***P < 0.001.

We evaluated the incidences of somatic mutations in 67 CRGs in patients with BLCA (**Figure 1A**). Out of 411 patients from the TCGA-BLCA cohort, 188 (45.74%) harbored mutations in CRGs. Of which, the frequency of mutations in *COL11A1* (6%) was the highest, followed by *FN1* and TNC (both 5% each). Nearly 4% of patients harbored mutations in *PDGFRA* and *MET* each and 3% in *ZFB1, COL3A1*, and *COL1A2*. Next, we determined CNV in CRGs, and the results revealed the highest number of CNV gains in *CTSK, S100A4*, and *CTHRC1* in patients with BLCA (**Figure 1B**). The chromosomal location of CNV in CRGs in patients with BLCA is shown in **Figure 1C**. The comprehensive picture of CRGs interactions and their prognostic value for BLCA was explored with a network (**Figure 1D**). The interaction between CRGs and their prognostic value in BLCA was shown in the network diagram. The results revealed a significant correlation between many CRGs and the patient’s prognosis. Finally, we determined CRGs expression in tissues of normal and patients with BLCA, and the results showed differential expression in 45 out of 67 CRGs (**Figure 1E**). An increase in *CD24, COL11A1, CTHRC1, CTS, FXYD3, HSPA1A, JUP, KRT7, MAN2B1, MET, MMP11, MMP9, PLAU, RAB3B, SPINT2*, and *TCN1* expression level was observed. The results revealed a decrease in *ACTA2, BEX5, CAT, CAV1, CD55, COL6A1, COL6A2, CTSK, EGR1, EMILIN1, FOS, FOXF1, GEM, HBA2, ID2, JUP, MFAP4, MFAP5, MMP2, OGN, PDGFRA, PDGFRB, PDPN, RARRES2, SLC16A4, TCEAL1, TNC*, and *ZEB1* expression level. These results show significant differences in the genetic alterations and the expression of CRGs in normal and tumor tissue, indicating significant involvement of CRGs in BLCA progression.

### 4.2 Identification of CAFs subtypes and their biological characteristics in patients with BLCA

**Figure 2:**
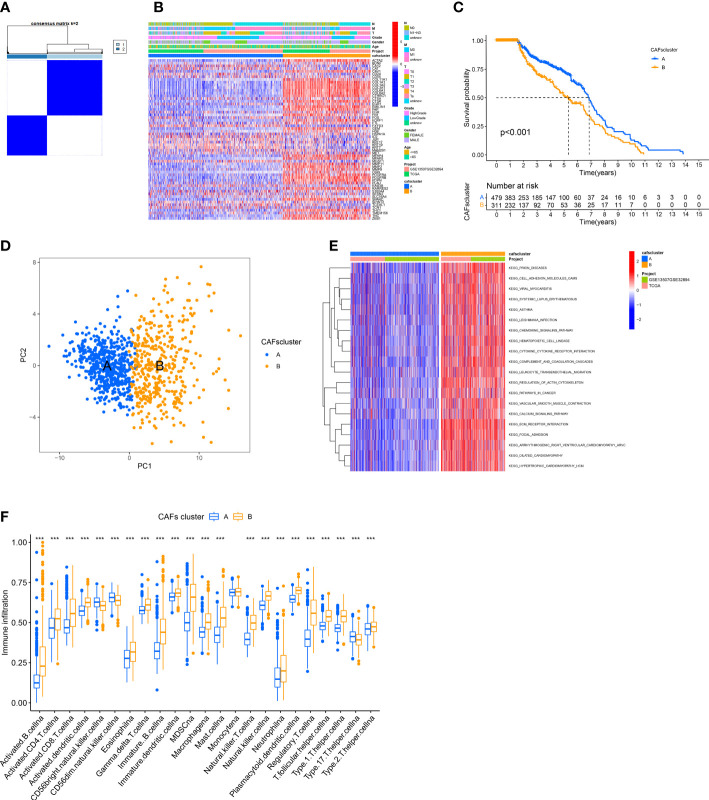
Identification of CAFs Subtypes and their Biological Characteristics in patients with BLCA. **(A)** The consensus clustering analysis was used to classify patients into two subtypes (k =2). **(B)** The heatmap shows the correlation between clinical characteristics and two subtypes. **(C)** KM survival curves were used for comparing the prognosis among patients in the two subtypes. **(D)** PCA of two clusters. **(E)** GSVA shows the pathways enriched in the two subtypes. **(F)** Infiltration of immune cells in the two subtypes. ***P < 0.001.

We merged the TCGA-BLCA cohort, GGSE13507, and GSE32894 into an entire cohort containing 958 patients. We performed consensus clustering based on 67 CRGs expressions to categorize all patients into two subtypes, cluster A containing 557 patients and cluster B containing 401 (**Figure 2A**). The heatmap shows the expression profile of 67 CRGs and the clinical characteristics of patients. The results revealed that the clinical characteristics of patients like gender, age, tumor grade, and metastasis were enriched in cluster B (**Figure 2B**). The prognostic analysis revealed that the survival status of patients in cluster A was better compared to cluster B (*P* < 0.001; **Figure 2C**). Principal component analysis (PCA) showed a significant transcription profile distribution into two groups (**Figure 2D**).

Next, we determined the difference in the biological functions enriched by the two clusters using GSVA. The results revealed significant enrichment of pathways linked to ECM production and remodeling like “CELL ADHESION MOLECULES,” “REGULATION OF ACTIN CYTOSKELETON,” “ECM RECEPTOR INTERACTION,” and “FOCAL ADHESION” in Cluster B (**Figure 2E**). Finally, we employed the “ssGSEA” algorithm to determine the proportion of tumor-infiltrating immune cells. The results revealed significant infiltration of activated lymphocytes in tumors of patients in cluster B, specifically activated CD4+ T cells, B cells, CD8+T cells, and monocytes. Moreover, high infiltration of T helper 17 (Th17) cells, as well as CD56 bright and CD56 dim natural killer cells, was observed in patients in cluster A (**Figure 2F**).

### 4.3 Characteristics of differently expressed CAFs-related genes

**Figure 3:**
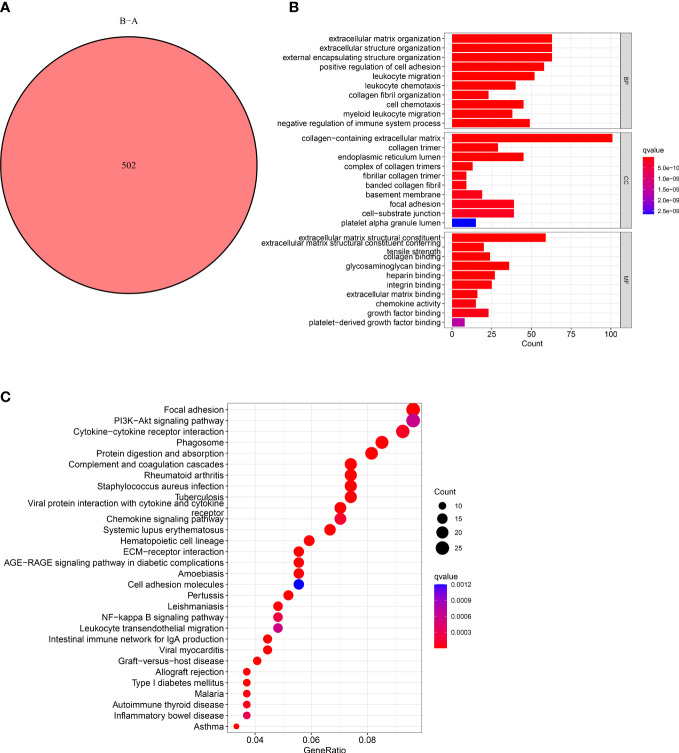
Characteristics of CAFs-Related DEGs. **(A)** Venn diagram shows pairwise DEGs in patients in the two subtypes. **(B)** The barplot graph shows the enrichment of GO analysis and CRGs. **(C)** The bubble graph shows the enrichment of KEGG pathways and CRGs.

The functions and pathways related to CAFs of the two subtypes were determined using the “limma” R package. We used these criteria: “|log2FC| ≥ 1” and “FDR <0.05” and identified 502 DECRGs associated with two subtypes (**Figure 3A**). Next, the “GO” and “KEGG pathway enrichment analyses” was conducted on 502 DECRGs. The “GO enrichment analysis” results showed that these CRGs were mainly enriched in the GO-BP terms like the positive regulation of ECM, extracellular structure, and external encapsulating structure organization. The GO-CC terms significantly enriched by these CRGs were collagen-containing ECM. Additionally, the significantly enriched GO-MF terms by these CRGs were the structural constituents of EC (**Figure 3B**). Meanwhile, the “KEGG pathway enrichment analysis” demonstrated that these CRGs were enriched in pathways related to focal adhesion (**Figure 3C**). Overall, these results suggest that CAFs mediate the invasion of cancer cells by depositing and modifying the ECM and promote tumor development by inducing epithelial-to-mesenchymal transition (EMT).

### 4.4 Construction and validation of the CAF-related genes prognostic model

**Figure 4:**
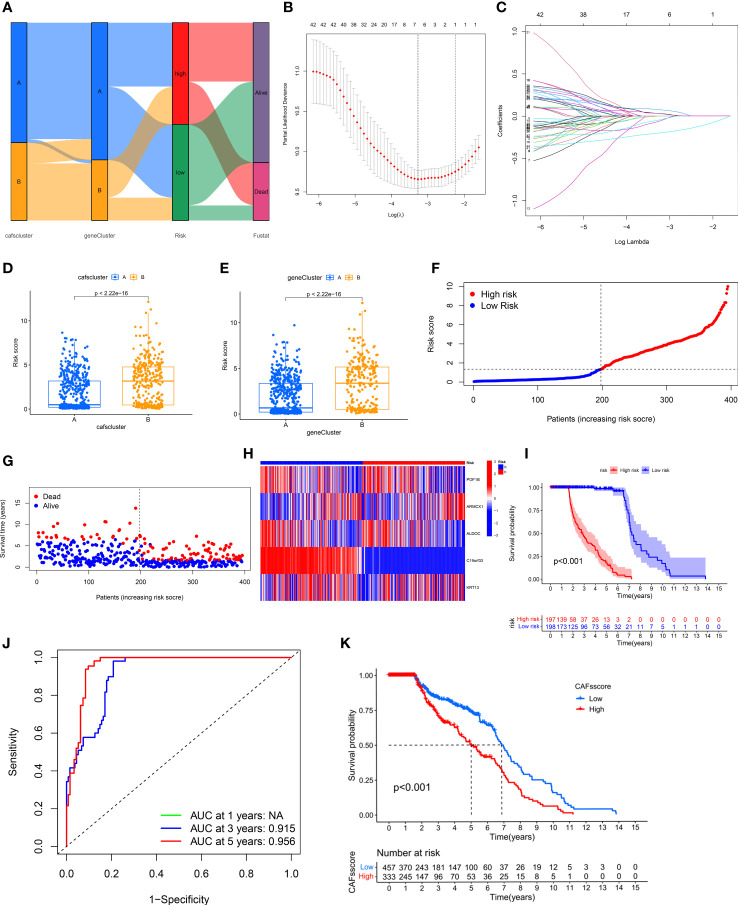
Construction and Validation of CRG Prognostic Model. **(A)** Sankey diagram shows the distribution of two CAFs subtypes. **(B, C)** LASSO regression analysis was performed on CRGs. **(D, E)** Significant differences in gene clusters and CAF clusters in patients in both risk groups. **(F, G)** The risk score plots of the two risk groups in the training set. **(H)** The risk heatmap of the two risk groups in the training set. **(I)** KM survival curves of the OS of patients in both risk groups in the training set (*P* < 0.001). **(J)** The ROC curves of 3- and 5-year OS of patients in both risk score groups in the training set. **(K)** CRGs-risk score for predicting the OS of patients with BLCA.

We calculated the CRGs-risk scores based on the DECRGs. The Sankey diagram shows the distribution of two CAFs subtypes, gene clusters, CRGs-risk scores, and the status of the patient’s survival (**Figure 4A**). Next, the entire set was randomly divided into the training (n=395) and testing (n=395) sets. Finally, we performed the “LASSO regression analysis” on 502 DEGs to identified and five CRGs (as prognostic gene signatures) (**Figures 4B, C**). Additionally, we performed a “multivariate Cox regression” analysis on these CRGs. Finally, five CRGs, including *POF1B, ARMCX1, ALDOC, C19orf33*, and *KRT13*, were identified, including two high-risk CRGs: *ARMCX1* and *KRT13*, as well as three low-risk CRGs: *POF1B, ALDOC*, and *C19orf33*. We calculated CRGs-risk scores based on the following formula: CRGs-risk scores = (-0.1622) * *POF1B* + (0.2061) * *ARMCX1* + (-0.2425) *ALDOC+ (-0.4558) * *C19orf33* + (0.0657) * *KRT13*.

The results showed significant differences in terms of CRGs-risk scores between the two clusters. The CRGs-risk scores of cluster A were low, and that of cluster B was high (**Figure 4D**). **Figure 4E** shows the distribution of CRGs-risk scores in the two subtypes.

Next, we categorized the patients based on the median CRGs-risk scores in high-risk group and low-risk group. The results showed a positive correlation between the mortality rate and the CRGs-risk scores (**Figures 4F, G**). Furthermore, the CRGs-risk scores heatmap showed an increase in *ARMCX1* and *KRT13* expression with an increase in risk scores (**Figure 4H**). In the training set, a significant difference in the survival of patients in both groups was observed (**Figure 4I**, *P* < 0.001). Moreover, the “receiver operating characteristic (ROC) curve” was constructed to determine the ability of CRGs-risk scores to predict patients’ prognoses. The AUC value of 3-year OS was 0.915, and the value of 5-year OS was 0.956 (**Figure 4J**). The prognosis of patients in low-risk group was significantly better compared to high-risk group, thus indicating the outstanding ability of CRGs-risk scores in predicting patients’ prognosis in BLCA (**Figure 4K**). Therefore, CRGs-risk scores could be an excellent model for predicting patients’ prognoses in BLCA.

### 4.5 Verification of the CAF-related genes prognostic model in the entire and test sets

**Figure 5:**
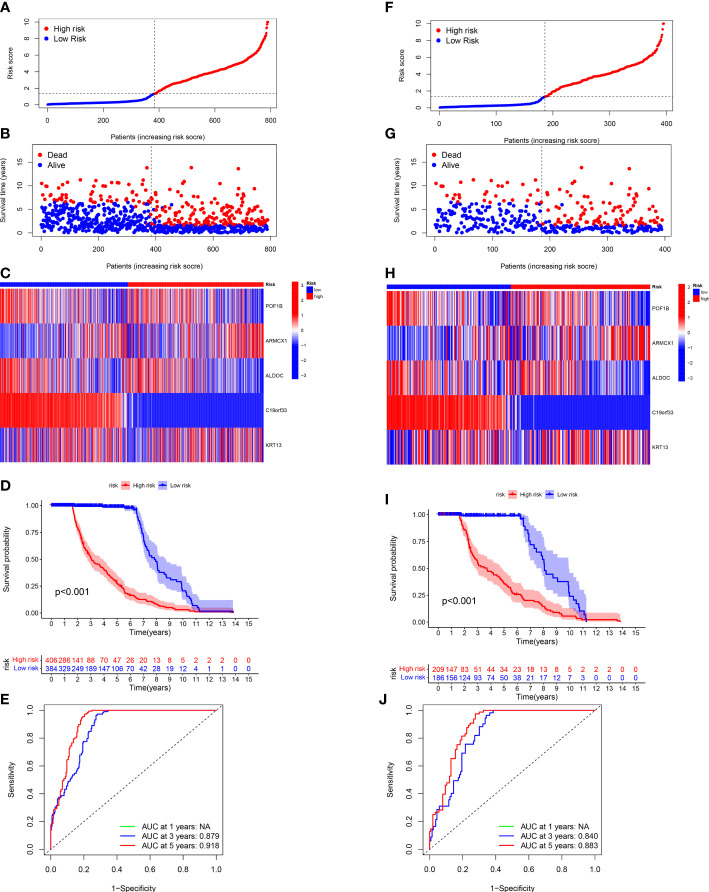
Validating the CRG Prognostic Model in the Entire and Test Sets. The risk plots, Survival duration and profanity, Risk heatmap, and ROC curves of 3- and 5 years for the risk score in the entire **(A-E)** and testing **(F-J)** sets.

Next, we validated the sensitivity, specificity, and ability of the CRGs prognostic model in predicting the prognosis of patients in the entire (**Figures 5A-D**) and test (**Figures 5I**) sets. The training set’s results were in line with our previous results. The ROC curves were used for predicting the sensitivity and specificity of 3- and 5-year OS rates of patients with different risk scores in the entire (**Figure 5E**) and test (**Figure 5J**) sets. The AUC values of 3- and 5-year OS of patients in the entire set were 0.879 and 0.918, respectively, and in the testing set were 0.840 and 0.883, respectively.

### 4.6 Assessment of TME of patients in both groups

**Figure 6:**
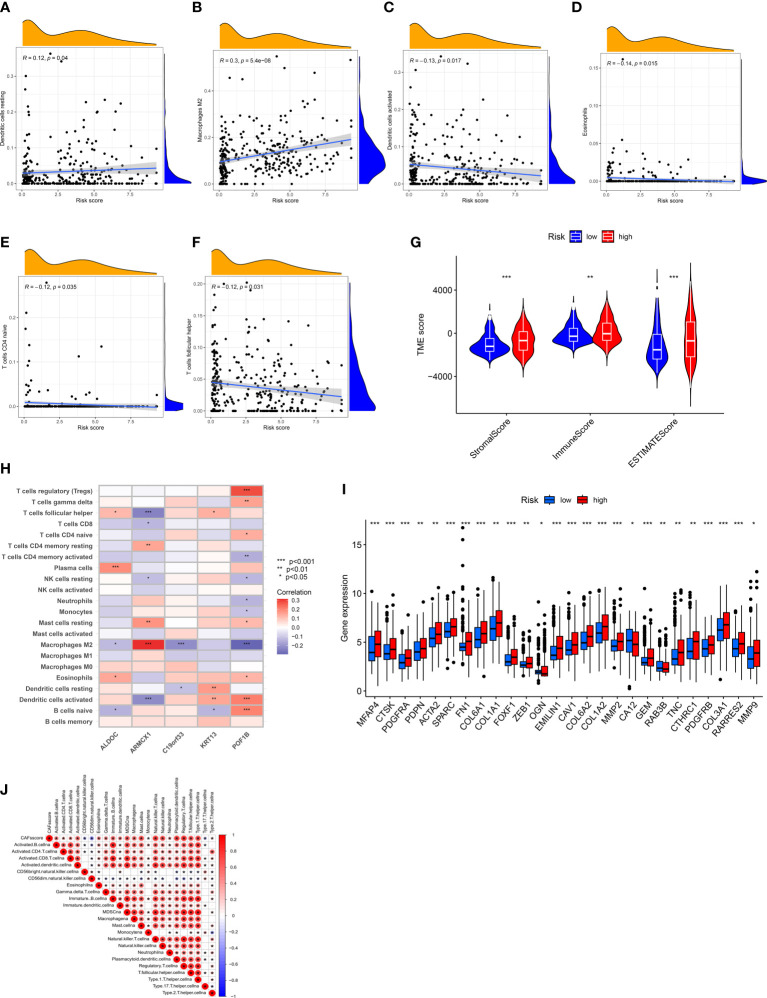
Assessment of TME of patients in Both Risk Groups. **(A–F)** A positive correlation between the CRGs-risk score and the resting DCs and M2 Macrophages. A negative correlation between the CRGs-risk score and the activated DCs, Eosinophils, naïve CD4 T cells, and Tfh cells. **(G)** Correlation between CRGs-risk score, immune, and stromal scores. **(H)** Correlation between five selected CRGs and the proportion of immune cells. **(I)** CRGs expression in patients in two risk groups. **(J)** Correlation between the CRGs-risk score and infiltrating immune cells. *P < 0.05, **P < 0.01, ***P < 0.001.

We employed the “CIBERSORT” algorithm to determine the correlation between the CRGs-risk scores and the proportion of immune cells. A positive correlation was observed between resting dendritic cells (DCs) as well as M2 macrophages and high CRGs-risk scores. A negative correlation between the high CRGs-risk scores and activated DCs, eosinophils, naive CD4 T cells, and T follicular helper (Tfh) cells (**Figures 6A-F**). Next, we calculated the stromal, ESTIMATE, and immune scores of patients with BLCA to determine TME status. The results revealed that the stromal, ESTIMATE, and immune scores of patients in high-risk group were significantly higher compared to patients in low-risk group. These results suggest an abundant relative concentration of stromal or immune cells in bladder tumor microenvironment (**Figure 6G**). We constructed the prognostic model based on five CRGs and evaluated the correlation between the proportion of immune cells and these five CRGs. The results revealed a significant correlation between these five CRGs and various immune cells (**Figure 6H**). Next, we evaluated the correlation between CRGs and the prognostic model. We determined the CRGs expression profile, and a significant difference in most CRGs expression was observed in patients in two risk groups. Additionally, an increase in *HGF, MMP11, OGN, RAB3B, KRT17*, and *BDNF* expression was observed in patients in low-risk group, whereas an increase in the expression of other CRGs was observed in patients in high-risk group (**Figure 6I**). Furthermore, we performed ssGSEA on infiltrating immune cells in TME to determine the alterations in the pathways (**Figure 6J**). The results showed a significant negative correlation between CAF-risk score and the abundance of activated immune cells against tumors like CD56 bright and CD56 dim natural killer cells, monocytes, and Th17 cells.

### 4.7 Evaluation of the correlation between CRGs-risk score and CSC, mutations, and drug sensitivity

**Figure 7:**
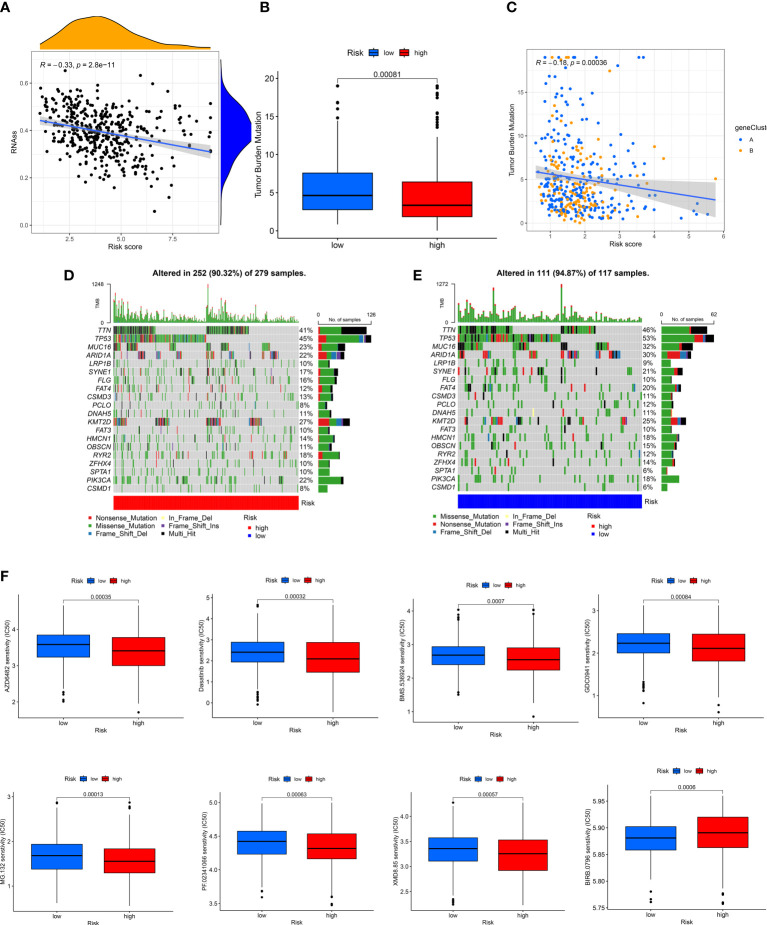
Evaluation of the correlation between CRGs-risk score and CSC, Mutation, and Drug sensitivity. **(A)** A positive correlation between the CSC index and CRGs-risk score. **(B, C)** The Boxplot and Spearman correlation shows that patients in LRH had a higher TME rate. **(D, E)** The somatic mutation in CRGs in patients in both groups. **(F, G)** The sensitivity of patients in both groups to chemotherapy drugs.

A positive linear correlation between CRGs-risk score and CSC index was observed (**Figure 7A**, R = -0.33, *P* < 0.001), suggesting that patients in low-risk group had higher stem cell-like characteristics. We analyzed the status of TMB in patients in the TCGA-BLCA cohort, and the results showed low TMB in patients in high-risk group compared to low-risk group (**Figure 7B**, *P* < 0.001). The “Spearman correlation analysis” revealed a negative correlation between the CRGs-risk score and TMB of patients (**Figure 6C**, R = -0.18, *P* < 0.001). Next, we analyzed the distribution of the somatic mutations in two risk groups from TCGA-BLCA cohort. The results revealed that the TOP10 mutated genes in patients in both risk groups were *TP53, TTN, MUC16, ARID1A, PCLO, LRP1B, FLG, SYNE1, FAT, CSMD3*, and *DNAH5* (**Figures 7D, E**). Compared to patients in high-risk group, the frequencies of mutations were higher in genes like *TTN, MUC16, TP53, ARID1A, PCLO*, *SYNE1*, and *FAT* and lower in genes like *LRP1B, FLG*, and *CSMD3* in patients in low-risk group (**Figures 7D, E**). Finally, we determined the correlation between the CRGs-risk score and chemotherapy drugs. Higher sensitivity of patients in low-risk group to chemotherapy drugs like AZD6482, BMS536924, Dasatinib, GDC0941, MG132, PF02341066, and XMD8.85 was observed (**Figure 7F**). However, the IC_50_ values of drugs like BIRB0796 were significantly higher in patients in high-risk group (**Figure 7G**).

### 4.8 Determining the expression CAF-related genes in tissues of normal bladder and BLCA by IHC images

**Figure 8:**
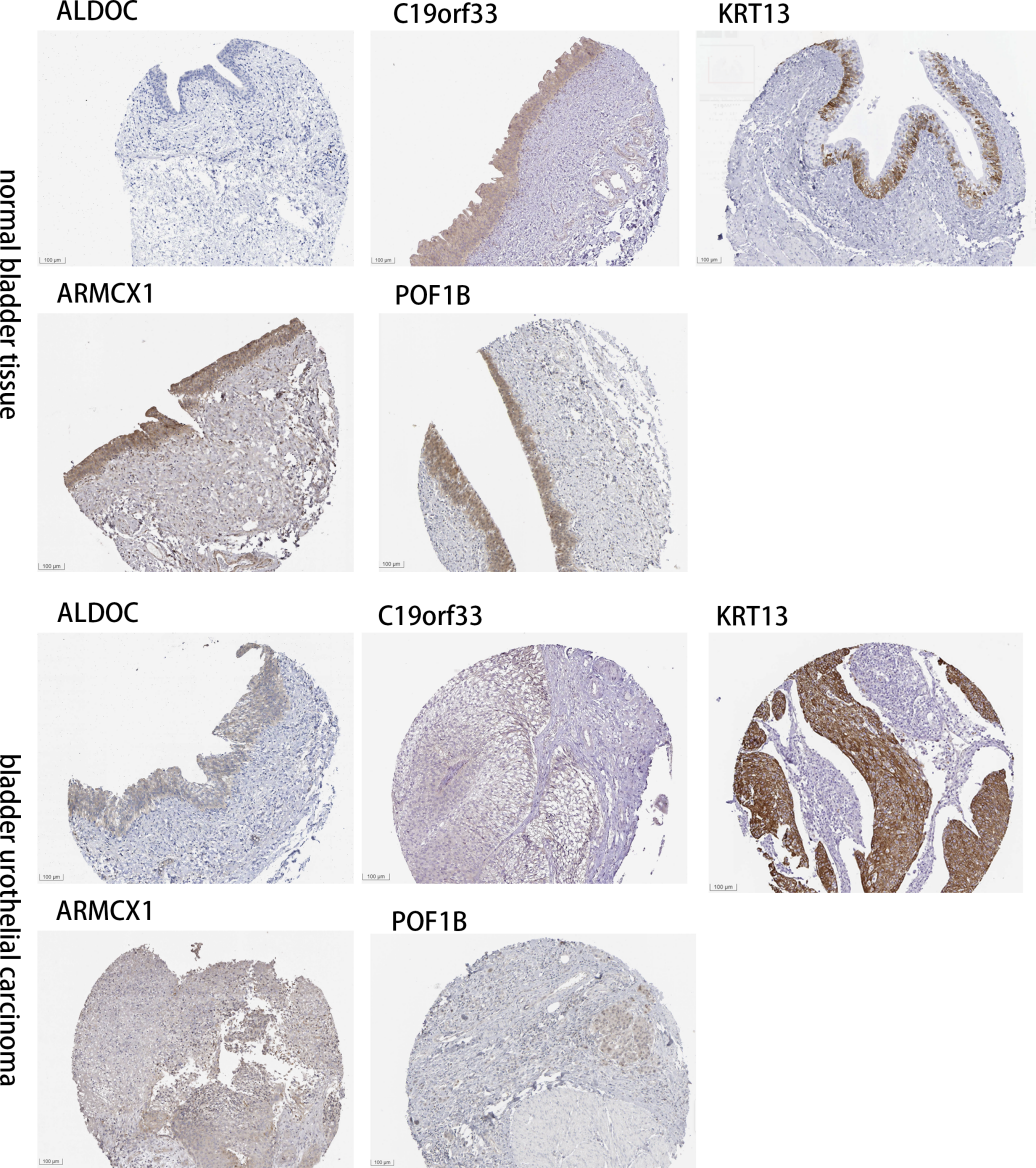
Determining the expression CRGs in tissues of normal bladder and BLCA by IHC images.

The “Human Protein Atlas” database was searched to validate the difference in CRGs expression between tissues of normal bladder and BLCA (**Figure 8**). Compared to normal bladder tissues, an increase in ARMCX1 and KRT13 expression levels was observed in BLCA. Additionally, a significant increase in POF1B, C19orf33, and ALDOC expression levels was observed in normal bladder tissues.

## 5 Discussion

A study has shown the significant influence of TME on BLCA onset and progression (19), and the heterogeneity of TME poses a challenge for the treatment of patients with BLCA (6). CAFs are the most abundant and widely studied stromal cells in TME and have a significant role in tumor progression, specifically solid tumors. CAFs are regulatory cells promoting cancer progression; hence, they could significantly contribute to the prognosis and treatment of patients with cancers (10). Several studies have reported that CAFs promote BLCA progression by enhancing matrix metalloproteinase (MMP) levels, which causes ECM deposition and modification (20). Furthermore, CAFs stimulate the formation of new vessels by secreting pro-angiogenic factors like platelet-derived growth factor (PDGF) and vascular endothelial growth factor (21). Additionally, CAFs secrete anti-inflammatory cytokines like IL-6 to induce EMT (22) and chemotactic factors like stromal cell-derived factor-1 to induce chemoresistance (23). CAFs are spindle-shaped cells, which express several mesenchymal markers, and lack non-mesenchymal cell markers, like epithelial, immune, endothelial, and neuronal cells (7). Chen et al. showed that Fibroblasts in BLCA could be divided into two different subgroups (myCAFs and iCAFs) by single-cell RNA sequencing on 8 BLCA samples. Comparing the iCAF and mCAF role in bladder tumor and found iCAF may be more involved in tumor progression and angiogenesis, tumor migration than mCAF (24).

In this study, we constructed a CRGs prognostic model for patients with BLCA. First, we determined somatic mutations and CNVs in 67 CRGs in patients with BLCA. The results revealed significant alterations in CRGs in these patients. Further, we determined the expression of 67 CRGs in tissues of patients with BLCA and normal, and the results demonstrated significant differences in the expression of 45 CRGs in BLCA and normal tissues. Furthermore, all patients were classified into clusters A and B by means of consensus clustering based on CRGs expression, and the differences in the two subtypes were determined. The prognostic analysis results showed higher survival, significant immune activation, and immune pathways in patients in cluster A compared to cluster B. Furthermore, the “GO” and “KEGG pathway enrichment analyses” showed that these CRGs were primarily enriched in functions like collagen-containing ECM, a structural constituent of ECM, and pathways related to focal adhesion. These results suggest that CAFs could induce EMT and modify ECM to promote tumorigenesis. Moreover, we determined DECRGs in patients in clusters A and B and performed “LASSO” and “multivariate cox regression” analyses. Finally, we identified five key CRGs like *POF1B, ARMCX1, ALDOC, C19orf33*, and *KRT13*, to construct the CRGs prognostic model. Based on the median CRGs-risk score, we classified all patients with BLCA into two CRGs-risk score groups. The results revealed significant differences in parameters like the patient’s prognosis, mutations, TME and CSC indexes, and drug sensitivity in both risk groups. Finally, we constructed a CRGs-risk score to explore BLCA characteristics. Meanwhile, our CRGs-risk score demonstrated good performance in predicting the OS and immune cell infiltration of patients with BLCA. We also used CRGs-risk score to investigate the dynamic changes in CAFs during tumorigenesis and BLCA progression, thereby indicating significant involvement of CAFs in tumor development in response to the TME.

Previous studies have explored the roles of five key CRGs included in our prognostic model. Of these five CRGs, two CRGs indicate poor prognosis. Studies have shown that KRT13 is localized in the suprabasal layers of the non-cornified stratified squamous epithelium of tonsils, the transitional urothelium, esophagus, larynx, esophagus, prostate tubule-initiating cells, and oral cavity (25, 26). Studies have shown that *KRT13* interacts with several proteins for regulating various signaling networks associated with the survival, death, migration, proliferation, invasion, and metastasis of cancer cells (27). Li et al. showed that KRT13 promotes the growth and metastasis of breast cancer cells via the plakoglobin/c-Myc pathway (28). Citron et al. showed that low KRT13 expression activates miR-9 to promote EMT in Neck Squamous Cell Carcinomas (29). Armadillo repeat-containing X-linked 1 (*ARMCX1*) is an arm protein lost in epithelial cancer on chromosome X1. It is involved in several cellular activities, like the growth and apoptosis of cells conforming to adhesion (30). A significant reduction in *ARMCX1* expression level was observed in tumor tissues compared to healthy samples from TCGA and GEO (31). Tang et al. showed that ARMCX1 could significantly inhibit gastric gancer onset via the mechanism of affecting the PAR-1/Rho GTPase pathway (32).

Additionally, a positive correlation was observed between the remaining three CRGs and the patient’s prognosis. *ALDOC* belongs to the aldolase family of isoenzymes and is critically involved in glycolysis and fructolysis (33). A study has shown a significant correlation between high ALDOC expression and longer survival duration of patients with advanced oral squamous cell carcinoma (34). Yuan et al. showed that the binding of ALDOC to GSK-β leads to the β-Catenin complex collapse and destruction, thus increasing cytoplasmic and nuclear levels of β-catenin in patients with non-small-cell lung cancer (35). POF1B was located in the critical region for normal ovarian function and participated in encoding premature ovarian failure (36). Studies predicting the functions of proteins have shown that POF1B had a tight homology relationship with the myosin tail portion of the human myosin protein (37). *C19orf33* has four exons spanning nearly 1 kb and 11 kb downstream of *HAI-2* (37). Studies have shown aberrant *C19orf33* expression in several cancers, including pancreatic cancer, and is closely related to the patient’s prognosis (38). Wen et al. showed that *C19orf33* inhibits breast cancer and papillary thyroid carcinoma progression by regulating EMT or YAP1 coordination in the Hippo pathway, respectively (39).

We have determined and validated the clinical significance of CAF-specific markers and constructed a CRGs prognostic model for patients with BLCA. However, our study has a few limitations. First, we have not used algorithms for deconvoluting CAFs in bulk RNA-seq to identify subgroups in an accurate manner. Second, our results are based on bioinformatics algorithms and should be validated in *in vivo* or *in vitro* models to understand the underlying mechanisms of CAFs in BLCA. Third, our CRGs prognostic model should be further validated using prospective clinical trials.

## 6 Conclusion

In summary, we constructed a CRGs prognostic model based on CAFs in patients with BLCA and identified five key prognostic genes like *POF1B, ARMCX1, ALDOC, C19orf33*, and *KRT13.* Our model could predict drug sensitivity and determine the immune status of patients with BLCA. Our results are helpful and can aid future studies on CAFs in BLCA.

## Data availability statement

The original contributions presented in the study are included in the article/**Supplementary Material**. Further inquiries can be directed to the corresponding author.

## Author contributions

MZ designed the study and drafted the manuscript. JZ and TL obtained and analyzed the data. MM prepared tables and figures. WH, PZ and LL supervised and revised the manuscript. All authors contributed to the article and approved the submitted version.
